# Achieving prediabetes reversal in China: a nationwide longitudinal study on the role of blood glucose and lipid management in middle-aged and elderly adults

**DOI:** 10.3389/fendo.2024.1463650

**Published:** 2025-01-22

**Authors:** Hongguang Yang, Yao Liu, Zhenhe Huang, Guifang Deng

**Affiliations:** ^1^ Department of Clinical Nutrition, Shenzhen Nanshan People’s Hospital, Shenzhen, Guangdong, China; ^2^ Geriatric Medicine Department, Shenzhen Nanshan People’s Hospital, Shenzhen, Guangdong, China

**Keywords:** prediabetes, normal glucose regulation, cohort, metabolic disorder, middle-aged and elderly adults

## Abstract

**Background:**

Prediabetes, impacting a third of the adult Chinese population, is linked to a variety of detrimental health outcomes. However, scant research has delved into the factors that affect a regression from prediabetes to normal glucose regulation (NGR) in middle-aged and elderly Chinese adults.

**Methods:**

We conducted a longitudinal analysis of 2,655 adults, aged 45 years and above, drawing data from wave 1 and wave 3 of the China Health and Retirement Longitudinal Study (CHARLS). We employed a stepwise logistic regression model to identify factors associated with the regression to NGR. Restricted Cubic Spline (RCS) analysis was used to evaluate the dose-response relationships between baseline fasting plasma glucose (FPG) and glycated hemoglobin (HbA1c) levels and the likelihood of regression to NGR. Attribution fraction (AF) analysis was conducted to measure the impact of modifiable factors on the regression of prediabetes. We further examined how changes in these factors were associated with regression to NGR.

**Results:**

During the 4-year follow-up, 570 of 2,655 prediabetes participants regressed to NGR. The stepwise logistic regression model identified older age, female sex, abdominal obesity (OR 0.70, 95% CI 0.57–0.86), elevated LDL-C (OR 0.69, 95% CI 0.48–0.97), higher FPG (OR 0.68, 95% CI 0.52–0.90), and higher HbA1c (OR 0.23, 95% CI 0.18–0.30) as factors associated with regression to NGR. AF analysis showed that a lower initial HbA1c was the most influential factor for regression to NGR. Additionally, evaluated blood lipid profiles reduced the odds of regression to NGR.

**Conclusion:**

This study underscores the influence of age, gender, abdominal obesity, LDL-C levels, FPG, HbA1c, and blood lipid profiles on the likelihood of regressing from prediabetes to NGR. It suggests that adopting a healthy lifestyle and preemptively mitigating these risks may be more beneficial than addressing them after they have been identified in clinical settings.

## Introduction

Prediabetes, a transitional phase between normal glucose regulation (NGR) and Type 2 diabetes mellitus (T2DM) (1), is characterized by blood glucose levels that exceed the normal range but fall short of the diagnostic thresholds for diabetes (2–4). Global prevalence estimates suggest that approximately 374 million adults suffer from impaired glucose tolerance (IGT), a form of prediabetes, with projections indicating this number could exceed 470 million by 2030 (1). In China, the estimated prevalence of prediabetes among adults was a staggering 38.1% in 2018, with the rate escalating alongside increasing age (5).

Similar to T2DM, prediabetes is associated with heightened risks for diabetes, cardiovascular diseases (6), stroke, chronic kidney diseases, and all-cause mortality (7). Evidence has shown that a reversal from prediabetes to NGR can significantly mitigate these health risks (8–10). For instance, in the Diabetes Prevention Program Outcome Study (DPPOS), participants who achieved NGR at least once during the study period experienced a 56% reduced risk of developing T2DM, compared to those who remained in a prediabetic state (9, 11). Additionally, prediabetes patients who successfully reverted to NGR saw a decrease in the risk of cardiovascular risk (10), microvascular disease, nephropathy and retinopathy (8).

Identifying the factors that facilitate the regression from prediabetes to NGR is crucial for improving health outcomes. Several studies have investigated these factors, with a DPPOS-based study highlighting the influence of age, gender, intensive lifestyle interventions, insulin secretion, baseline blood glucose levels, triglycerides, and weight loss on this regression (9, 12). Another cohort study corroborated the positive impact of weight loss on prediabetes reversal, although the effects of baseline body mass index (BMI) and waist circumference (WC) remain inconsistent (13).

However, most previous research has concentrated on American or European populations, with racial and regional differences in the pathophysiological mechanisms of prediabetes and T2DM suggesting the need for more diverse studies (14–16). There is a dearth of research focusing on the Chinese population. Moreover, it is estimated that China has the largest number of diabetes patients globally, with over a third of adults in the country having prediabetes (5). Therefore, identifying and intervening on the factors that influence the regression from prediabetes to NGR in the Chinese population can significantly improve the health status of Chinese adults and potentially reduce the incidence of prediabetes and diabetes, thereby alleviating the economic and social burden associated with these conditions.

Therefore, the aim of this study is to investigate the factors linked to the regression of prediabetes to NGR in Chinese population using data from China Health and Retirement Longitudinal Study (CHARLS). Such research is essential for enhancing health outcomes for individuals with prediabetes.

## Materials and methods

### Study population

Data from the CHARLS were used. The details of the program’s objectives, design and methods can be found elsewhere (17). The CHARLS is a prestigious, ongoing, and nationally representative longitudinal survey. The project employed a multi-stage sampling approach, utilizing the probability proportional to size sampling method at both the county/district and village/community sampling stages. Its purpose is to meticulously collect a robust set of high-quality microdata that reflects the lives of households and individuals in China who are 45 years of age and above. The inaugural national baseline survey was launched in 2011, reaching into 450 villages, spanning 150 counties within 28 provinces, and engaging over 17,000 individuals. This benchmark study is meticulously repeated every 2 to 3 years to provide a snapshot of the demographic trends. The data is meticulously collected through face-to-face interviews. CHARLS collected data on body measurements and blood samples. The datasets could be acquired at http://charls.pku.edu.cn/en once the online application was approved and five national waves data are available including the baseline survey (2011y, wave 1), the first followed data (2013y, wave2), second follow‐up survey (2015y, wave 3), third follow‐up survey (2018y, wave 4), and fourth follow‐up survey (2020y, wave 5). The CHARLS project was approved by the Biomedical Ethics Committee of Peking University, and all participants were required to sign informed consents.

**Figure 1:**
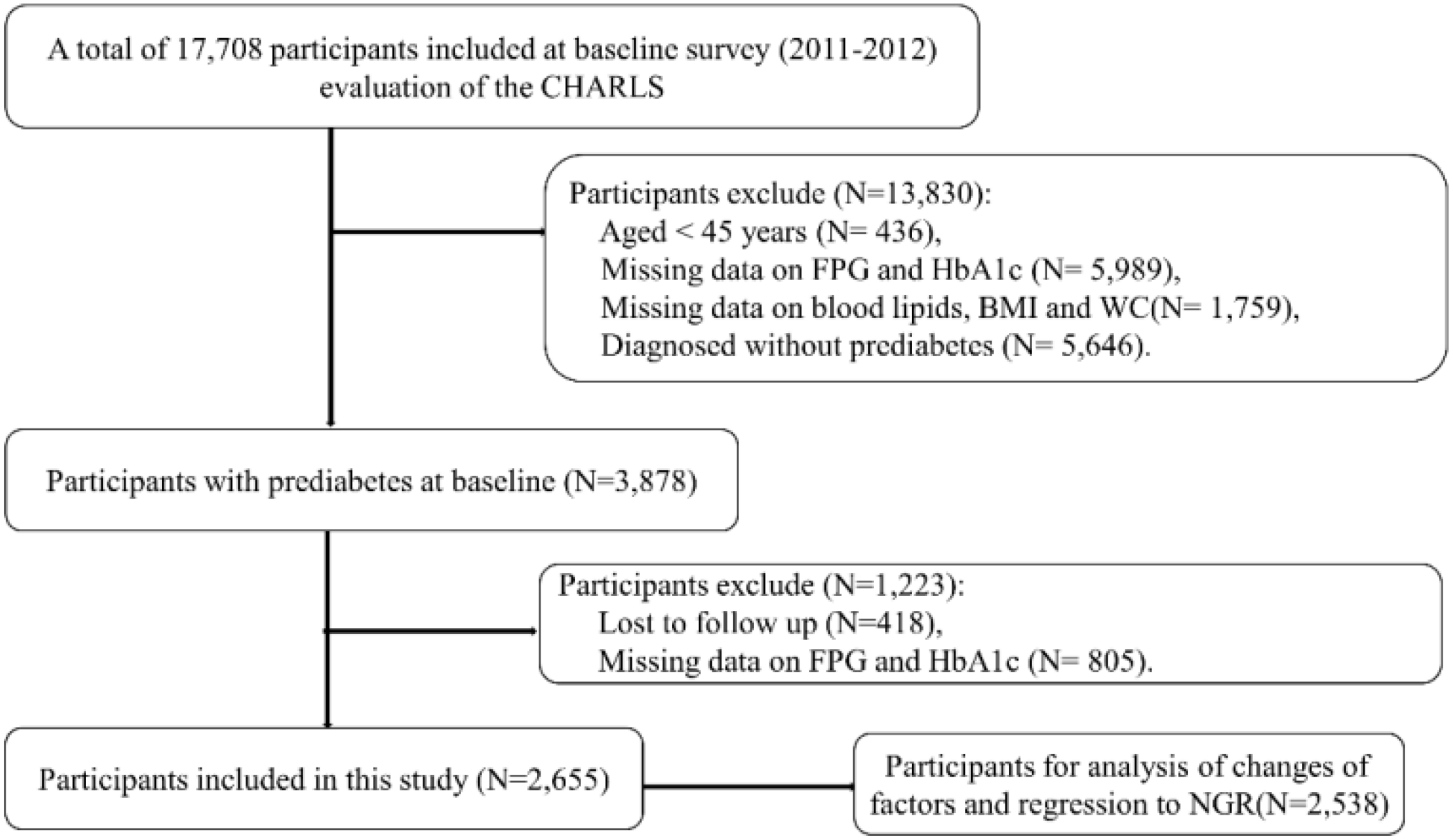
Flowchart of the study population.

The data used in this study was from wave 1 and wave 3. **Figure 1** presented the procedures of the study design, sampling and exclusion. Of 17,708 participants at baseline, a total of 2,655 participants with prediabetes were included in the present study.

### Data assessment

#### Data collection and measurements

Fasting blood samples were collected by trained staff and transported to the designated laboratory for bioassays. FPG, TG, total cholesterol (TC), low -density lipoprotein cholesterol (LDL-C), and high-density lipoprotein cholesterol (HDL-C) were measured by enzymatic colormetric test. HbA1c and uric acid (UA) were measured by boronate affinity HPLC and UA plus method, respectively.

Body height, weight and WC were measured by trained staff according to the research protocol. The BMI was calculated by dividing body weight (kg) by body height squared (m^2^).

#### Definitions of glycemic status

According to the ADA criteria (18), prediabetes was defined as FPG in the range of 5.6~6.9 mmol/L and/or HbA1c in the range of 5.7~6.4%; diabetes was as FPG ≥ 7.0mmol/L, HbA1c ≥ 6.5%, self-reported history and/or the use of anti-diabetic medications, and NGR was as FPG <5.6 mmol/L and HbA1c < 5.7%.

#### Covariates

In this study, we took into account a range of sociodemographic, behavioral, and health characteristics that could potentially confound our results, drawing on established knowledge.

Our sociodemographic assessment encompassed age, gender (female and male), educational attainment categorized as primary school or below, middle school, and high school or above, the distinction between rural and urban residences, and marital status which included categories for married and unmarried/divorced individuals.

Behavioral factors considered included drinking habits, with participants classified as non-drinkers or drinkers, and smoking status, categorized as non-smokers or smokers.

Health characteristics were also a focal point, with a particular emphasis on obesity status and the history of chronic diseases such as dyslipidemia and hypertension. Obesity was further divided into general and abdominal obesity. General obesity was identified by a BMI of 28 kg/m² or higher (19), while abdominal obesity was determined by a waist circumference of at least 85 cm for females and 90 cm for males (19). Hypertension was determined as either clinical diagnosis, hypotensive drug treatment, SBP ≥ 140 mmHg and/or DBP ≥ 90 mmHg (20). Dyslipidemia was defined as TC ≥ 6.22 mmol/L, TG ≥ 2.26 mmol/L, HDL-C < 1.04 mmol/L, LDL-C ≥ 4.14 mmol/L and/or self-reported dyslipidemia (21). Additionally, blood lipid parameters were stratified into low and high levels using the aforementioned cut-off values.

### Statistical analysis

Continuous variables in this study were presented as means ± standard deviations and compared by t-test. Categorical variables were presented as frequency (n, %) and compared by chi-square test. Multiple logistic regression analysis was conducted to explore the association between factors which showed significant difference between two groups with prediabetes regression to NGR. Stepwise regression based on the Akaike information criterion (AIC) minimum was used to select optimal feature factors as the previous research (14).

To elucidate the dose-response relationship between fasting plasma glucose (FPG) and glycated hemoglobin (HbA1c) levels in relation to the regression to NGR, we utilized Restricted Cubic Spline (RCS) analyses. These analyses were instrumental in identifying FPG and HbA1c as critical determinants of prediabetes status.

As part of our sensitivity analysis, we employed a multinomial logistic regression model. This model accounted for three distinct glycemic outcome groups observed during the follow-up period: regression to NGR, progression to diabetes, and remained as prediabetes. For the purposes of this analysis, the group that remained prediabetic at the follow-up was used as the reference category.

Attribution fraction (AF) was used to evaluate the contribution of the modifiable factors for regression to NGR. AF was the embodiment of the percentage reduction of a given outcome that was expected if there was no exposure (22). AFs and 95% CI were calculated with the ‘AF’ R package (22). In addition, individual AFs cannot be summed directly to obtain an overall AF as given the assumption that the independence of risk factors is likely biased (23) and the individual AFs may be over-evaluated.

We conducted a further analysis to assess the correlation between changes in modifiable risk factors and the regression to NGR. This evaluation involved calculating the changes in these variables by determining the difference between their follow-up values and their initial baseline measurements.

All statistical analyses were performed using SPSS version 24.0 (IBM Corp., Armonk, NY) and R (version 4.2.2, http://www.r-project.org). A two-sided *p* value < 0.05 was considered statistically significant.

## Results

### Baseline characteristics

The baseline characteristics of the participants were detailed in **Table 1**. Among 2,655 participants identified with prediabetes at baseline, 570 (21.5%) reverted to NGR over the subsequent 4-year follow-up period. In comparison with those who did not revert to NGR, individuals who achieved NGR were younger, predominantly male, and exhibited lower levels of TC, LDL-C, fasting plasma glucose, HbA1c, and WC, as well as a reduced prevalence of general and abdominal obesity (all p-values < 0.05). When participants were categorized into three distinct groups (regression to NGR, progression to diabetes, and remained as prediabetes), similar patterns in baseline characteristics were observed across all groups (**Supplementary Table S1**).

**Table 1:** Baseline characteristics of participants stratified by glycemic outcomes.

Variables		Total (N=2655)	Regressed to NGR		*P*
			No (N=2085)	Yes (N=570)	
Age, year		59.4 ± 8.7	59.7 ± 8.7	58.3 ± 8.8	**<.001**
Gender, n (%)	Male	1216 (45.8)	923 (44.3)	293 (51.4)	**0.003**
	Female	1439 (54.2)	1162 (55.7)	277 (48.6)	
Educational levels, n (%)	Primary school and lower	1903 (71.7)	1516 (72.7)	387 (67.9)	**0.042**
	Middle school	545 (20.5)	418 (20)	127 (22.3)	
	High and above	207 (7.8)	151 (7.2)	56 (9.8)	
Places of residence, n (%)	Rural	2218 (83.5)	1737 (83.3)	481 (84.4)	0.582
	Urban	437 (16.5)	348 (16.7)	89 (15.6)	
Drinking, n (%)	No	1791 (67.5)	1420 (68.1)	371 (65.1)	0.189
	yes	864 (32.5)	665 (31.9)	199 (34.9)	
Smoking, n (%)	No	1010 (38.0)	783 (37.6)	227 (39.8)	0.347
	yes	1645 (62.0)	1302 (62.4)	343 (60.2)	
Marital status, n (%)	married	2320 (87.4)	1820 (87.3)	500 (87.7)	0.840
	Not married	335 (12.6)	265 (12.7)	70 (12.3)	
Hypertension, n (%)		1124 (42.3)	902 (43.3)	222 (38.9)	
Dyslipidemia, n (%)		960 (36.2)	774 (37.1)	186 (32.6)	0.054
Uric acid, mg/dL		4.5 ± 1.2	4.5 ± 1.2	4.4 ± 1.3	0.532
TG, mmol/L		1.5 ± 1.0	1.5 ± 0.9	1.5 ± 1.0	0.593
TC, mmol/L		5.1 ± 1.0	5.2 ± 1.0	4.9 ± 1.0	**<.001**
HDL-C, mmol/L		1.3 ± 0.4	1.3 ± 0.4	1.3 ± 0.4	0.955
LDL-C, mmol/L		3.1± 0.9	3.1 ± 0.9	2.9 ± 0.9	**<.001**
FPG, mmol/L		6.0 ± 0.4	6.0 ± 0.4	6.0 ± 0.3	**0.011**
HbA1c, %		5.2 ± 0.4	5.3 ± 0.4	5.0 ± 0.4	**<.001**
WC, cm		85.0 ± 12.4	85.6 ± 12.4	83.0 ± 12.2	**<.001**
BMI		23.8 ± 3.8	23.9 ± 3.8	23.3 ± 3.8	**0.001**
Abdominal obesity, n (%)		1197 (45.1)	997 (47.8)	200 (35.1)	**<.001**
General obesity, n (%)		330 (12.4)	278 (13.3)	52 (9.1)	**0.009**

NGR, normal glucose regression; TG, triglycerides; TC, total cholesterols; LDL-C, low-density lipoprotein cholesterols; HDL-C, FPG, fasting plasm glucose; HbA1c, glycated hemoglobin; high-density lipoprotein cholesterols; WC, waist circumference, BMI, body mass index.

Bold font indicates P<0.05.

### Associated factors and regression to NGR

The associations between baseline characteristics and regression to NGR were presented in **Table 2**. In the stepwise logistic regression model, variables including age, gender, abdominal obesity, LDL-C, FPG, and HbA1c were identified as significant predictors of the regression from prediabetes to NGR. The results indicated that older age (OR, 0.98; 95%CI, 0.97 to 0.99), female sex (OR, 0.79; 95%CI, 0.65 to 0.97), abdominal obesity (OR, 0.70; 95%CI, 0.57 to 0.86), higher LDL-C (OR, 0.69; 95%CI, 0.48 to 0.97), higher FPG (OR, 0.68; 95%CI, 0.52 to 0.90), and higher HbA1c (OR, 0.23; 95%CI, 0.18 to 0.30) were associated with a reduced likelihood of reverting to NGR.

**Table 2:** Multivariate logistic regression analysis for prediabetes regression to NGR.

Variables		Model 1	Model 2
		OR (95%CI)	OR (95%CI)
Age		0.98 (0.97,0.99)	**0.98 (0.97,0.99)**
Gender	Male	Reference	Reference
	Female	0.80 (0.65,0.98)	**0.79 (0.65,0.97)**
Educational levels	Primary school and lower	Reference	
	Middle school	0.95 (0.74,1.22)	
	High school and above	1.17 (0.82,1.66)	
Abdominal obesity		**0.71 (0.57,0.88)**	**0.70 (0.57,0.86)**
General obesity		0.95 (0.67,1.34)	
LDL-C	Low	Reference	Reference
	High	0.79 (0.50,1.25)	**0.69 (0.48,0.97)**
TC	Low	Reference	
	High	0.82 (0.53,1.26)	
FPG		**0.69 (0.52,0.91)**	**0.68 (0.52,0.90)**
HbA1c		**0.23 (0.18,0.30)**	**0.23 (0.18,0.30)**

The same as **Table 1**. OR, odd ratio; CI, confidence interval. Model 1 was multivariate logistic regression model. Model 2 was the stepwise logistic regression model based on Model 1. Blanks in Model 2 represented variables that were statistically nonsignificant and were excluded from the final model.

Bold font indicates P<0.05.

The sensitivity analyses based on multinomial logistic regression model did not influence the primary analysis outcomes (**Table 3**).

**Table 3:** Multinomial logistic regression analysis based on three glycemic outcomes for prediabetes regression to NGR.

Variables	Regressed to NGR (N=570)	Remained as prediabetes (N=1667)	Progressed to T2DM (N=418)
	OR (95%CI)	OR (95%CI)	OR (95%CI)
Age	**0.98(0.97,0.99)**	Reference	**1.02(1.00,1.03)**
Gender (female)	**0.79(0.64,0.96)**	Reference	0.95(0.75,1.19)
Abdominal obesity	**0.78(0.63,0.96)**	Reference	**1.88(1.49,2.37)**
High LDL-C	**0.68(0.48,0.97)**	Reference	0.97(0.70,1.35)
FPG	0.77(0.58,1.03)	Reference	**1.99(1.50,2.64)**
HbA1c	**0.27(0.21,0.34)**	Reference	**2.42(1.83,3.19)**

The same as **Table 1**. OR, odd ratio; CI, confidence interval.

Bold font indicates P<0.05.

**Figure 2:**
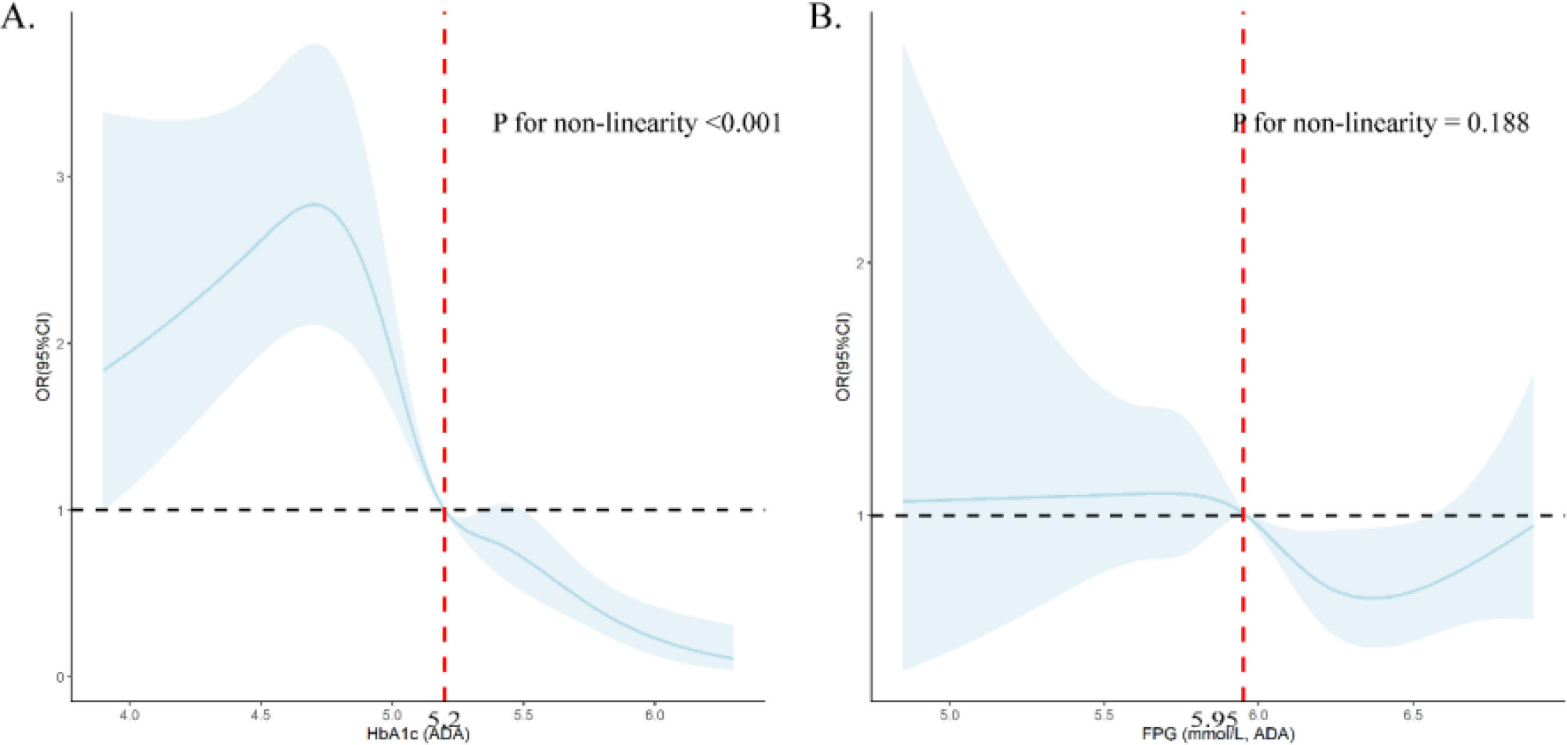
Dose-response relationship between HbA1c **(A)** and FPG **(B)** and prediabetes regression to NGR using restricted cubic splines (RCS). **(A)** was adjusted for age, gender, abdominal obesity, LDL-C level and FPG; **(B)** was adjusted for age, gender, abdominal obesity, LDL-C level and HbA1c.

The dose-response relationship evaluated by RCS models showed nonlinear relationships between FPG and HbA1C and regression to NGR (**Figure 2**).

### AF analysis of modifiable factors associated with regression to NGR

The AFs of modifiable factors associated with regression to NGR were presented in **Table 4**. After adjusting for covariates, individuals with low HbA1c levels (<5.2%) were found to have the highest AF for regression to NGR, at 46.0% (95%CI, 38.5% to 53.5%). This was followed by those with low LDL-C levels (<4.14 mmol/L), with an AF of 22.7% (95%CI, 3.0% to 42.3%), absence of abdominal obesity with an AF of 14.60% (95%CI, 6.2% to 23.0%), and low FPG levels (<5.95 mmol/L) with an AF of 11.20% (95%CI, 4.1% to 18.2%).

**Table 4:** AFs of modifiable factors associated prediabetes regression to NGR.

Exposure	Adjusted-AF	95%CI	p-value
No abdominal obesity	14.60%	(6.2%,23.0%)	**<0.001**
Low LDL-C	22.70%	(3.0%,42.3%)	**0.024**
Low FPG	11.20%	(4.1%,18.2%)	**0.002**
Low HbA1c	46.00%	(38.5%,53.5%)	**<0.001**

AF, attribution fraction; CI, confidence interval. The model included age, gender, abdominal obesity, LDL-C, FPG and HbA1c. The model for each exposure was adjusted for all other factors. Low LDL-C, baseline LDL-C < 4.14 mmol/L; Low FPG, baseline FPG < 5.95 mmol/L; Low HbA1c, baseline HbA1c < 5.2%.

Bold font indicates P<0.05.

### Changes of modifiable factors and regression to NGR

The associations between changes of modifiable factors and regression to NGR were presented in **Table 5**. After adjusting for covariates, elevated levels of LDL-C (OR, 0.71; 95%CI, 0.60 to 0.85), TC (OR, 0.72; 95%CI, 0.62 to 0.83), and TG (OR, 0.85; 95%CI, 0.75 to 0.96) were inversely associated with the likelihood of regression to NGR. In contrast, changes in BMI, WC, and HDL-C did not show a significant association with the regression to NGR.

**Table 5:** Changes of modifiable factors and prediabetes regression to NGR.

Variables	Model 1	Model 2
	OR (95%CI)	OR (95%CI)
WC change	1.004 (0.997,1.012)	0.998 (0.991,1.007)
BMI change	0.98 (0.94,1.01)	0.97 (0.93,1.01)
LDL-C change	0.92 (0.80,1.05)	**0.71 (0.60,0.85)**
TC change	**0.75 (0.65,0.86)**	**0.72 (0.62,0.83)**
TG change	**0.89 (0.90,0.99)**	**0.85 (0.75,0.96)**
HDL-C change	0.83 (0.60,1.16)	0.82 (0.53,1.26)

The same as **Table 1**. OR, odd ratio; CI, confidence interval. Model 1 was adjusted for age, gender, abdominal obesity status, LDL-C, FPG and HbA1c at baseline. For WC change, except abdominal obesity status and for LDL-C change, except baseline LDL-C. Model 2 was further adjusted the according variable at baseline.

Bold font indicates P<0.05.

## Discussion

In our study, we delved into the factors that might influence the regression from prediabetes to NGR. Stepwise logistic regression models revealed that a combination of unmodifiable (age and gender) and modifiable factors (including abdominal obesity, blood lipid levels, FPG, and HbA1c) were significantly and independently linked to the likelihood of regression to NGR.

### Estimated rate of prediabetes regression to NGR

In the present study, 570 (21.5%) participants with prediabetes at baseline regressed to NGR. The estimated rate of prediabetes regression to NGR in this study was consistent with our previous study using CHARLS (24), but diverged from other previous studies, which reported rates ranging from 9.3% to 27.3% (13, 14, 25). For example, the German study reported 9.3% of participants regressed from glucose-defined prediabetes to NGT, and 27.3% from HbA1c-defined prediabetes to normal HbA1c (13). Moreover, during 10 years of follow-up, 17.1% of participants in Japan (25) and 36% in Korea regressed from prediabetes to NGT (14). These studies utilized varying criteria for defining prediabetes, with disparities in the study populations and durations of follow-up periods. Therefore, a large-scale, multi-regional study adhering to standardized criteria is essential to accurately estimate the prevalence and modifiable factors influencing the regression from prediabetes to NGR, thereby enhancing clinical insights.

### Unmodifiable factors and prediabetes regression

Aging was widely acknowledged to be associated with prediabetes and T2DM. Consistent with the majority of previous studies (9, 12, 26), older age was one of the most important factors that decreased odds of regression to NGR in our study. The aging process adversely affects β-cell function, exacerbating the deficiency in insulin production and heightening insulin resistance, potentially through mechanisms such as obesity, reduction in pancreatic parenchymal volume, and other contributing risk factors (27–30). These physiological changes collectively increased the risk of prediabetes and reduced the chances of regression to NGR (27–30).

The role of gender in prediabetes regression remained mixed. A DPPOS study demonstrated that female sex increased odds of regression from prediabetes to isolated IFG and decreased odd of regression to isolated IGT (9), another two follow-up studies reported no significant effect of gender (14, 31). The present study also showed that being female decreased the odds of regression to NGR, possibly due to the decline in estrogen levels in menopausal and postmenopausal women. Estrogen plays a pivotal role in augmenting insulin sensitivity and in promoting the secretion and utilization of insulin (32, 33). Previous studies indicated that following menopause, estrogen levels in women decrease with age, resulting in impaired glucose metabolism and an increased risk of developing diabetes (32, 33).

### Modifiable factors and prediabetes regression

In addition to the unmodifiable factors of age and gender, our study identified several modifiable predictors that are significantly associated with the regression to NGR. Specifically, we found that blood glucose levels, obesity, and lipid profiles were independently linked to the likelihood of reverting from prediabetes to NGR. Therefore, from the perspective of public health prevention, individuals with prediabetes can promote the regression to NGR by controlling these modifiable factors, thereby preventing complications and improving the quality of life.

Previous studies demonstrated that baseline glucose status was a main predictor for regression to NGR (9, 14, 31, 34). Studies from Sweden (31), Korea (14), and America (9) demonstrated that both FPG and 2-h glucose were associated with regression to NGT. An Australian cohort study focusing on women reported that FPG levels were linked to a reduced probability of reverting to normoglycemia over a decade-long follow-up period (34). Our study corroborated these findings, indicating a significant association between FPG and regression to NGR. However, the relationship between HbA1c and regression to NGR has been less explored. Our study revealed a strong association between HbA1c levels and the regression to NGR. Notably, a lower initial HbA1c level was found to be the most influential factor in promoting regression to NGR. It’s worth noting that HbA1c can effectively reflect an individual’s blood glucose level over the past three months (35), independent of several factors including whether fasting before the test and the use of insulin or metformin (36), and smaller biological within‐subject variation for than FPG (37). As such, HbA1c may emerge as a pivotal prognostic indicator for the regression to NGR from prediabetes in clinical practice. It is important for individuals with prediabetes to regularly monitor their HbA1c levels, with the optimal target being below 5.2%.

Previous research had reported inconsistent findings on the link between baseline BMI and WC and the regression to NGR from prediabetes. For example, Kowall et al. found that baseline BMI and WC were associated with regression from HbA1c-based or glucose-based prediabetes to HbA1c-based or glucose-based normoglycemia, respectively (13), while Paprott et al. did not (26). Our study emphasized that abdominal obesity, more than general obesity, hindered regression to NGR. Abdominal obesity and glucose metabolism disorder was related to ectopic adipose tissue (EAT) more than subcutaneous adipose tissue (SAT) (38, 39). Even in individuals of normal weight, an excess of EAT can elevate the risk of insulin resistance (IR) and impair insulin secretion (38). The flow of non-esterified fatty acids produced by excess abdominal fat to the liver may impair hepatic metabolism and lead to increased hepatic glucose production (27, 39). Furthermore, an overabundance of EAT can trigger endocrine dysfunction, dysregulation of inflammatory factors, and a decrease in adiponectin levels, all of which contribute to IR (40).

Lipid levels have been implicated in the regression from prediabetes to NGR. A Korean 10-year follow-up study reported that high TG was against to regression to NGT (14). Similarly, an Australian 10-year cohort study identified high TG levels and their increase as having adverse effects on regression (17). Rebecca et al. reported that high HDL-C increased odds of regression to NGR (26). The present study reported that low LDL-C and reduced lipid levels contributed to regression to NGR. Lipid and glucose metabolism are intricately connected; excessive TG can induce inflammation and endoplasmic reticulum stress, impairing insulin activity, glucose uptake in muscles, and β-cell function (41, 42).

### Limitations and strengths

Some limitations in this study should be mentioned. Firstly, the definitions for glycemic outcomes were based on FPG and HbA1c, while the lack of data on oral glucose tolerance test due to the CHARLS design might cause diagnostic bias though a previous study demonstrated nearly 90% prediabetes could be identified by only FPG and HbA1c (43). Secondly, variables such as diet habits, drug use, and body fat, which might affect the glycemic outcomes, were not available in the CHARLS dataset, and their exclusion could potentially impact the final conclusions. Thirdly, FPG and HbA1c were from single measurements instead of repeated measurements in CHARLS sample measurements, which may bias the actual results. Fourthly, the study population were derived from CHARLS aged 45 and older, therefore the conclusions might not be generalizable to other populations.

This study also had strengths. First, the study population with a large size came from a nation-wide, representative and community-based population. Second, we tried to explore the factors associated with prediabetes regression to NGR among middle-aged and elder Chinese adults, and different models were used to verify the results.

## Conclusions

In conclusion, our study identified several factors linked to the regression from prediabetes to NGR, including older age, female sex, abdominal obesity, and dyslipidemia, along with elevated FPG and HbA1c levels. For individuals with prediabetes, the management of blood glucose and lipid profiles emerges as a pivotal clinical strategy. From a preventative standpoint, fostering a healthy lifestyle and mitigating these risks proactively may be more beneficial than reactive interventions post-detection.

## Data Availability

The original contributions presented in the study are included in the article/**Supplementary Material**. Further inquiries can be directed to the corresponding author/s.
