# Supplementary material for: Achieving prediabetes reversal in China: a nationwide longitudinal study on the role of blood glucose and lipid management in middle-aged and elderly adults
Source: Front Endocrinol (Lausanne). 2025 Jan 22;15:1463650. doi: 10.3389/fendo.2024.1463650 (PMC11794071; doi:10.3389/fendo.2024.1463650)
Supplement: Supplementary file 1 [file Table1.docx]

# Supplementary materials

Table S1 Baseline characteristics of participants stratified by three glycemic outcomes

| Variables |  | Regressed to NGR (N=570) | Remained as prediabetes (N=1667) | Progressed to T2DM (N=418) | *P* |
| --- | --- | --- | --- | --- | --- |
| Age, year |  | 58.3 ± 8.8 | 59.5 ± 8.6 | 60.8 ± 8.8 | <.001 |
| Gender, % | Male | 293 (51.4) | 744 (44.6) | 179 (42.8) | 0.008 |
|  | Female | 277 (48.6) | 923 (55.4) | 239 (57.2) |  |
| Educational levels, % | Primary school and lower | 387 (67.9) | 1202 (72.1) | 314 (75.1) | 0.075 |
|  | Middle school | 127 (22.3) | 345 (20.7) | 73 (17.5) |  |
|  | High school and above | 56 (9.8) | 120 (7.2) | 31 (7.4) |  |
| Places of residence, % | Rural | 481 (84.4) | 1394 (83.6) | 343 (82.1) | 0.615 |
|  | Urban | 89 (15.6) | 273 (16.4) | 75 (17.9) |  |
| Drinking, % | No | 371 (65.1) | 1124 (67.4) | 296 (70.8) | 0.165 |
|  | Yes | 199 (34.9) | 543 (32.6) | 122 (29.2) |  |
| Smoking, % | No | 227 (39.8) | 618 (37.1) | 165 (39.5) | 0.407 |
|  | Yes | 343 (60.2) | 1049 (62.9) | 253 (60.5) |  |
| Marital status, % | Married | 500 (87.7) | 1463 (87.8) | 357 (85.4) | 0.415 |
|  | Not married | 70 (12.3) | 204 (12.2) | 61 (14.6) |  |
| Hypertension, % |  | 222 (38.9) | 690 (41.4) | 212 (50.7) | <.001 |
| Dyslipidemia, % |  | 186 (32.6) | 594 (35.6) | 180 (43.1) | 0.003 |
| TG, mmol/L |  | 1.5 ± 1.0 | 1.5 ± 0.9 | 1.6 ± 1.0 | 0.012 |
| TC, mmol/L |  | 4.9 ± 1.0 | 5.1 ± 1.0 | 5.2 ± 1.0 | <0.001 |
| LDL, mmol/L |  | 2.9 ± 0.9 | 3.1 ± 0.9 | 3.2 ± 0.9 | <0.001 |
| HDL, mmol/L |  | 1.3 ± 0.4 | 1.3 ± 0.4 | 1.3 ± 0.4 | 0.003 |
| Uric acid, mg/dL |  | 4.4 ± 1.3 | 4.5 ± 1.2 | 4.6 ± 1.2 | 0.164 |
| BMI |  | 23.3 ± 3.8 | 23.7 ± 3.7 | 24.9 ± 4.1 | <0.001 |
| WC, cm |  | 83.0 ± 12.2 | 84.9 ± 12.4 | 88.6 ± 12.2 | <0.001 |
| Abdominal obesity, % |  | 200 (35.1) | 742 (44.5) | 255 (61) | <0.001 |
| General obesity, % |  | 52 (9.1) | 193 (11.6) | 85 (20.3) | <0.001 |
| FPG, mmol/L |  | 6.0 ± 0.3 | 6.0 ± 0.4 | 6.1 ± 0.4 | <0.001 |
| HbA1c, % |  | 5.0 ± 0.4 | 5.2 ± 0.4 | 5.4 ± 0.4 | <0.001 |

Abbreviations: NGR, normal glucose regression; TG, triglycerides; TC, total cholesterols; LDL-C, low-density lipoprotein cholesterols; HDL-C, FPG, fasting plasm glucose; HbA1c, glycated hemoglobin; high-density lipoprotein cholesterols; WC, waist circumference, BMI, body mass index.
